# Case Report: A Rare Case of Metachronous Multiple Primary Lung Cancers in a Patient With Successful Management by Switching From Anti-PD-1 Therapy to Anti-PD-L1 Therapy

**DOI:** 10.3389/fimmu.2021.683202

**Published:** 2021-06-02

**Authors:** Xinqing Lin, Guihuan Qiu, Fang Li, Haiyi Deng, Yinyin Qin, Xiaohong Xie, Juhong Jiang, Yong Song, Ming Liu, Chengzhi Zhou

**Affiliations:** ^1^ State Key Laboratory of Respiratory Disease, National Clinical Research Centre for Respiratory Disease, Guangzhou Institute of Respiratory Health, First Affiliated Hospital, Guangzhou Medical University, Guangzhou, China; ^2^ Department of Medical Center, Geneplus-Beijing, Beijing, China; ^3^ State Key Laboratory of Respiratory Disease, The First Affiliated Hospital of Guangzhou Medical University, Guangzhou, China; ^4^ Department of Respiratory and Critical Care Medicine, Jinling Hospital, Nanjing, China; ^5^ The First School of Clinical Medicine, Southern Medical University, Guangzhou, China

**Keywords:** metachronous multiple primary lung cancer (mMPLC), immunotherapy, resistance, next-generation sequencing (NGS), re-biopsy

## Abstract

Without global standard diagnostic criteria, distinguishing multiple primary lung cancers (MPLCs) from intrapulmonary metastasis or histologic transformation has been a big challenge in clinical practice. Here, we described a rare case of metachronous adenocarcinoma and small cell lung cancer (SCLC) in a patient who developed drug resistance to pembrolizumab. Both DNA-sequencing and RNA-sequencing were performed on primary adenocarcinoma and resistant lesions. Through the comparison of primary adenocarcinoma and novel lesion mutation profiles, along with bioinformatic estimation of immune proportion by using RNA sequence data, we revealed the origin and tumor microenvironment of the two lesions. No shared mutations were detected between lung adenocarcinoma (LUAD) and SCLC from the same patient, suggesting these two lesions might be from separate primary lung cancers. Compared to LUAD, SCLC showed a relatively cold microenvironment, including negative PD-L1. The patient obtained durable clinical benefits upon treatment with atezolizumab, without experiencing immune-related adverse events. Disease progression should be monitored with prompt re-biopsy and molecular profiling to spot a potential histologic change and to shed light on therapeutic alternatives. The use of atezolizumab, either alone or in combination with other agents, may be a potential therapeutic strategy for patients with both LUAD and SCLC.

## Introduction

Lung cancer is one of the most leading causes of cancer-related death worldwide (1). Metachronous multiple primary lung cancer (mMPLC) is an infrequent entity, with an incidence rate around 1~2%, with the majority sharing the same histologic type (2). Discriminating MPLCs from intrapulmonary metastasis especially inmultifocal lung cancers remains a major challenge to oncologists.

Recently, many immune checkpoint inhibitors (ICIs) have been approved for the treatment of advanced lung cancer. However, it is still unknown if ICIs are effective for MPLC patients, particularly those with different histology. We herein present a rare case of mMPLC in a patient with lung adenocarcinomas (LUAD) and small cell lung cancer (SCLC) during treatment with ICIs. Both genomic profiles and tumor microenvironment (TME) were evaluated to explore the inter-tumor heterogeneity and decipher the potential mechanisms behind checkpoint blockade in this patient.

## Case Report

**Figure 1:**
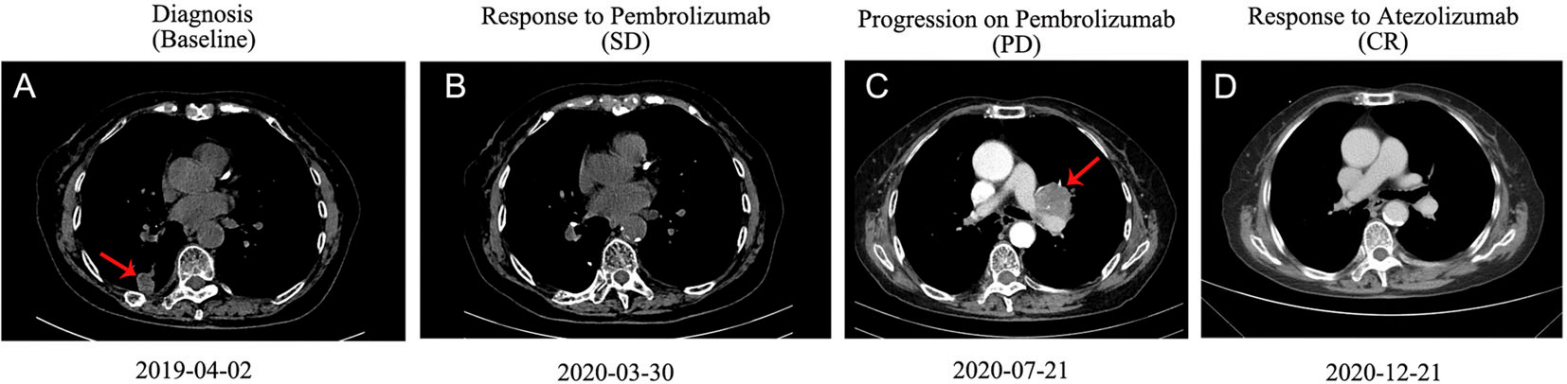
Computed tomography (CT) scans of the thorax of the patient. **(A)** CT scan before the treatment. **(B)** Image of the best response of pembrolizumab therapy. **(C)** Image of progression of pembrolizumab therapy and before atezolizumab therapy. **(D)** Image of best response of atezolizumab therapy. CR, complete response; SD, stable disease; PD, progressive disease.

**Figure 2:**
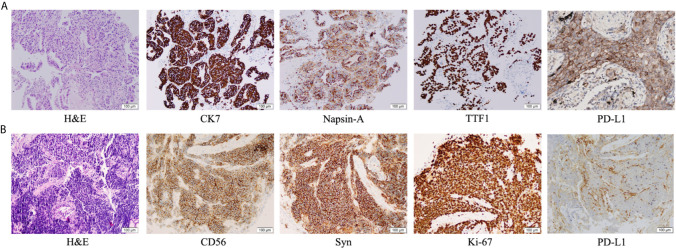
Immunohistochemical examination: **(A)** Pathologic findings from the right lower lobe lesion at the time of initial diagnosis showed adenocarcinoma (hematoxylin and eosin [H&E] staining), with positive immunohistochemical staining for CK7, Napsin-A, TTF1 and PD-L1. **(B)** Pathologic finding from the intraluminal lesion in the left bronchus at the time of progression showed small cell lung cancer (H&E), with positive immunohistochemical staining for CD56, synaptophysin and Ki-67, and negative staining for PD-L1.

**Figure 3:**
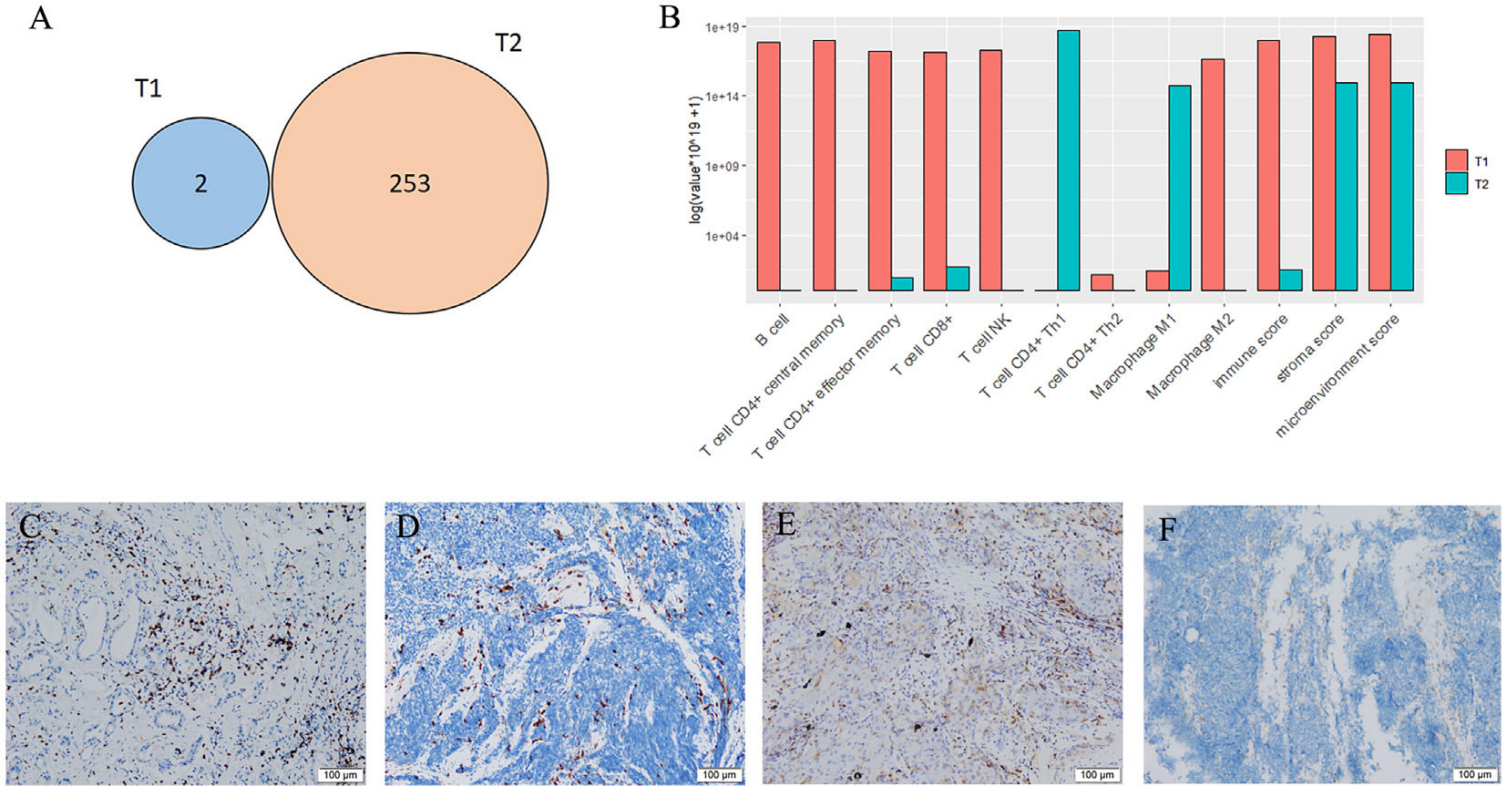
Mutations and tumor microenvironments. **(A)** Overlap of non-silent mutations of adenocarcinoma (T1) and small cell lung cancer (T2) samples from the same patient. **(B)** Fractions of infiltrating cells. **(C)** CD8^+^ T cells of T1. **(D)** CD8^+^ T cells of T2. **(E)** CD4^+^ T cells of T1. **(F)** CD4^+^ T cells of T2.

An 83-year-old woman with a history of 40 pack-year smoking and 30-year chronic obstructive pulmonary disease presented with shortness of breath, coughing with ever-increasing violence and expectoration for a month. The chest computed tomography scan was performed, which revealed a solid nodule in the right lower lung (**Figure 1A**). Multiple lymph node metastases were also observed in the right lung, right hilum, and right pleura. Serum tumor marker level was 15.8 ng/mL in neuron-specific enolase (NSE). She was performed the video-assisted thoracoscopic guided wedge resection of the right lower lung on April 24, 2019. Pathologic analysis of the postoperative specimens confirmed infiltrating adenocarcinoma (**Figure 2A**) and systemic work-up staged the tumour as cT4N2M1a. In addition, the tumor tissue (T1) acquired during operation was sent for genomic testing by capture-based targeted sequencing (520 cancer-related genes). Mutations in *NF1* and *MAX* were identified in T1, without other mutations. At that time, the biopsy specimen showed high PD-L1 expression with a tumor proportion score (TPS) of 80% by immunohistochemistry (Dako 22C3) (**Figure 2A**). The patient then received one cycle of pembrolizumab combined with recombinant human endostatin for malignant pleural effusion, after which she continued with pembrolizumab 200 mg every three weeks since June 2019 with a stable disease status (**Figure 1B**). Pembrolizumab treatment stopped in July 2020 when a newly emerged left hilar mass shadow was found in the patient (**Figure 1C**). And the NSE was elevated to 19.74 ng/mL. The combination of pemetrexed and bevacizumab was administered on July 23, 2020. Subsequently, the bronchoscopic biopsy sample from the left lung hilar mass showed tumor cells positive for synaptophysin, CD56, and Ki-67 (80%), which were consistent with SCLC, and negative for PD-L1 (**Figure 2B**). Whole exome sequencing (WES) was applied for the new lesion (T2), with a total of 253 non-silent somatic mutations detected, including a *TP53* S241C mutation at an allele frequency (AF) of 78.6%, a *RB1* K63Ifs*46 mutation with AF 64.3%, and no *NF1* mutation was detected. No shared mutations were detected between T1 (520-target panel sequencing) and T2 (WES) from the same patient (**Figure 3A**), and the two lesions contain different oncogenic driver mutations, suggesting that these two lesions might be from separate primary lung cancers. RNA-sequencing was also performed to investigate the tumor microenvironments in T1 and T2. Using single sample gene set enrichment analysis (ssGSEA), we estimated RNA-seq-derived infiltrating immune cell populations for these two samples. Compared with the “original” T1, T2 exhibited less immune infiltrating cells, including CD8^+^ T cells and B cells, and lower score of immune and microenvironment (**Figure 3B**). The different distributions of CD8^+^ and CD4^+^ T cells between two tissues were validated by immunohistology (**Figures 3C–F**). Due to ECOG PS of 2 and patient’s wish of no chemotherapy, she was switched to atezolizumab 1200mg every three weeks in July 2020. The patient experienced a complete response to atezolizumab based on the 5-month scan (**Figure 1D**). To date, this patient still accepts the PD-L1 inhibitor monotherapy with no immune-related adverse events and a sustained complete response.

## Discussion

Histologic transformation of non-small-cell lung cancer to SCLC has been reported to be a potential mechanism of resistance to immunotherapy (3, 4). Therefore, it is difficult to distinguish mMPLC from intrapulmonary metastasis or treatment-induced transformation. However, the advanced diagnostic methods in histopathological analyses and molecular characteristics analyses, particularly using next-generation sequencing (NGS), allow us to better diagnose mMPLCs. Some studies suggested that multiple tumors were independent if they had different driver mutations, and were metastatic if they shared gene mutations for even just one driver mutation in common (5–7). In our case, loss of the initial *NF1* driver mutation at T2 diagnosis and no *RB1* mutation was detected in T1 raise the possibility that T2 was a second primary malignancy. Notably, the genomic profiles of T1 and T2 from the same patient were completely different. Although a mixed histology at diagnosis should be considered due to limited specimen and inconsistent genes panel, elevated serum tumor marker, good response to initial therapy and less aggressive clinical course than SCLC supported the diagnosis of mMPLC.

Furthermore, using RNA-sequencing, we also found the inter-tumor heterogeneity in tumor microenvironment between T1 and T2. The distinct features of TME and genomic features between T1 and T2 mainly were associated with disparities in prognosis. In our patient, T2 was identified as SCLC with a high tumor mutation burden (TMB-H), which has been proposed as a predictive biomarker for response to ICI across multiple tumour types (8), largely due to potential for tumor mutations to generate immunogenic neoantigens. While, it has been recently reported that TMB-H does not predict response to ICI in cancer types where neoantigen load is not positively correlated with CD8 T cell levels (9). In this patient, a lower level of immune score and less CD8^+^ T cells infiltrating were found in T2 than T1. The immune and stromal score are derived from the immune contexture, and is defined by immune cell infiltration based on ssGSEA. Several studies have proved that higher immune/stromal score are associated with favorable prognosis of human malignancies, such as hepatocellular carcinoma, pancreatic cancer, melanoma, and lung cancer (10). It was suggested that insufficient immune infiltration of T2 might lead to failures in tumor immune surveillance and trigger tumor immune escape. Moreover, T2 has been shown with a lower intra-tumoral PD-L1 expression. Together, these findings may shed light on the resistance to pembrolizumab in SCLC.

The combined-histology lung cancers in the patient made the next treatment decision difficult. Recently, combination chemotherapy and ICIs has been reported to have good results in treating SCLC and has already changed the treatment algorithm of SCLC (11). However, no consistent predictive biomarkers that can accurately guide the use of ICIs in patients with SCLC have been identified, including the expression of PD-L1. The working hypothesis regarding why combination treatment has proved to be the most successful strategy to date is that chemotherapy administration results in increased presentation of tumour-associated antigens, resulting in increased T cell priming and amplification of the cytotoxic T cell response. The patient has received one cycle of pemetrexed and bevacizumab before the administration of atezolizumab. It is also supposed that antiangiogenic therapy could increase the infiltration of immune effector cells into tumors and convert the intrinsically immunosuppressive tumour microenvironment to an immunosupportive one (12). Remarkably, the CD4^+^ T helper 1 (Th1) cells were highly enriched in T2 (**Figure 3B**) before the treatment, which was reported as a predictive marker of response to PD-L1 or PD-1 targeted immunotherapies in multiple cancer types (13, 14). But we also found the expression levels of immunologically relevant genes secreted by Th1 cells, such as interferon-γ and IL2, were extremely low in T2 according to the data of RNA-sequencing, revealing the transcriptomic programs maybe not activated in the infiltrating immune cells. However, there are not available samples to confirm the intra-tumoral CD8^+^ T cells increase following combination treatment in the patient, as it has been shown in metastatic renal cell carcinoma (15). Further researches are warranted to explore the potential association about these features.

To our best knowledge, this is the first case about the second primary lung cancer responding well to a PD-L1 inhibitor after the resistance to PD-1 inhibitor. It is also the first report to use genome and transcriptome sequencing to analyze the origin and tumor microenvironment of metachronous LUAD and SCLC. In conclusion, re-biopsy and molecular profiling should be highlighted in routine clinical evaluation upon disease progression with current treatment.

## Data Availability Statement

The datasets presented in this study can be found in online repositories. The names of the repository/repositories and accession number(s) can be found in the article/**Supplementary Material**.

## Ethics Statement

Written informed consent was obtained from the individual(s) for the publication of any potentially identifiable images or data included in this article.

## Author Contributions

XL and GQ: data collection, data analysis, data interpretation and manuscript preparation. FL and HD: data analysis and data interpretation. YQ, XX, and JJ: data collection. YS, ML, and CZ: data review and interpretation. All authors contributed to the article and approved the submitted version.

## Funding

This work was supported by the Zhongnanshan Medical Foundation of Guangdong Province [ZNSA-2020003].

## Conflict of Interest

FL was employed by company Geneplus-Beijing Ltd.

The remaining authors declare that the research was conducted in the absence of any commercial or financial relationships that could be construed as a potential conflict of interest.
