# Supplementary material for: Case Report: A Rare Case of Metachronous Multiple Primary Lung Cancers in a Patient With Successful Management by Switching From Anti-PD-1 Therapy to Anti-PD-L1 Therapy
Source: Front Immunol. 2021 Jun 2;12:683202. doi: 10.3389/fimmu.2021.683202 (PMC8207139; doi:10.3389/fimmu.2021.683202)
Supplement: Supplementary file 1 [file DataSheet_1.docx]

**Supplementary Method**

**Whole-Exome Sequencing and analysis**

DNA was extracted from tumor tissue using QIAamp DNA FFPE Tissue Kit (Qiagen GmbH, Hilden, Germany), according to the manufacturer’s instructions. Peripheral blood lymphocytes (PBLs) were used to extract germline genomic DNA with the DNeasy Blood Kit (Qiagen, Valencia, CA). Indexed NGS libraries were constructed from sheared DNA using the DNA Library Preparation Kit for MGISeq-2000 (BGI, Shenzhen, China). The libraries were hybridized to SeqCap EZ Exome 64M (Roche NimbleGen, Madison, WI, USA). DNA sequencing was performed using the MGISeq-2000 Sequencing System (BGI, Shenzhen, China) with paired-end 100bp. Burrows–Wheeler Aligner (BWA) was employed to align the clean reads to the reference human genome (hg19). Single nucleotide variants (SNVs) and somatic small insertions and deletions (InDels) were called with GATK and MuTect. Copy-number variants (CNVs) were identified with CONTRA 2.0.8 software.

**Gene expression data analysis**

RNA was extracted from tumor tissue using QIAamp RNeasy FFPE Kit (Qiagen GmbH, Hilden, Germany). The mRNA libraries were prepared using the NEBNext® Ultra™ RNA Library Prep Kit for Illumina® (#E7530L, NEB, USA) according to the manufacturer’s protocol, RNA-seq libraries were paired-end sequenced on an MGISEQ-2000 sequencer. Sequencing reads containing adaptor sequences and low-quality reads were filtered. Cean reads were mapped to hg19 using STAR (v2.7.5). The single sample gene set enrichment analysis (ssGSEA) was used to calculate the enrichment scores (ES) of immune cell types in the tumor microenvironment. Immune cell infiltrations、immune score and stromal score were determined by Xcell R package (https://github.com/dviraran/xCell/blob/master/R/xCell.R). For each sample, expression levels of genes were normalized to gene length (TPM) and the final output is the adjusted xCell scores.
